# Supplementation with Live and Heat-Treated *Lacticaseibacillus paracasei* NB23 Enhances Endurance and Attenuates Exercise-Induced Fatigue in Mice

**DOI:** 10.3390/nu17152568

**Published:** 2025-08-07

**Authors:** Mon-Chien Lee, Ting-Yin Cheng, Ping-Jui Lin, Ting-Chun Lin, Chia-Hsuan Chou, Chao-Yuan Chen, Chi-Chang Huang

**Affiliations:** 1 Graduate Institute of Sports Science, National Taiwan Sport University, Taoyuan City 333325, Taiwan; kurt0710@ntsu.edu.tw (M.-C.L.); 1130203@ntsu.edu.tw (T.-Y.C.); 2 Center for General Education, Taipei Medical University, Taipei 110301, Taiwan; 3 New Bellus Enterprise Co., Ltd., Tainan 720008, Taiwan; billy@newbellus.com.tw (P.-J.L.); tony@newbellus.com.tw (T.-C.L.); chou@newbellus.com.tw (C.-H.C.); 4 Physical Education Office, National Taipei University of Business, Taipei 10051, Taiwan

**Keywords:** *Lacticaseibacillus paracasei* NB23, probiotics, postbiotics, fatigue, exercise performance

## Abstract

**Background**: Exercise-induced fatigue arises primarily from energy substrate depletion and the accumulation of metabolites such as lactate and ammonia, which impair performance and delay recovery. Emerging evidence implicates gut microbiota modulation—particularly via probiotics—as a means to optimize host energy metabolism and accelerate clearance of fatigue-associated by-products. **Objective**: This study aimed to determine whether live or heat-inactivated *Lacticaseibacillus paracasei* NB23 can enhance exercise endurance and attenuate fatigue biomarkers in a murine model. **Methods**: Forty male Institute of Cancer Research (ICR) mice were randomized into four groups (*n* = 10 each) receiving daily gavage for six weeks with vehicle, heat-killed NB23 (3 × 10^10^ cells/human/day), low-dose live NB23 (1 × 10^10^ CFUs/human/day), or high-dose live NB23 (3 × 10^10^ CFUs/human/day). Forelimb grip strength and weight-loaded swim-to-exhaustion tests assessed performance. Blood was collected post-exercise to measure serum lactate, ammonia, blood urea nitrogen (BUN), and creatine kinase (CK). Liver and muscle glycogen content was also quantified, and safety was confirmed by clinical-chemistry panels and histological examination. **Results**: NB23 treatment produced dose-dependent improvements in grip strength (*p* < 0.01) and swim endurance (*p* < 0.001). All NB23 groups exhibited significant reductions in post-exercise lactate (*p* < 0.0001), ammonia (*p* < 0.001), BUN (*p* < 0.001), and CK (*p* < 0.0001). Hepatic and muscle glycogen stores rose by 41–59% and 65–142%, respectively (*p* < 0.001). No changes in food or water intake, serum clinical-chemistry parameters, or tissue histology were observed. **Conclusions**: Our findings suggest that both live and heat-treated *L. paracasei* NB23 may contribute to improved endurance performance, increased energy reserves, and faster clearance of fatigue-related metabolites in our experimental model. However, these results should be interpreted cautiously given the exploratory nature and limitations of our study.

## 1. Introduction

Exercise-induced fatigue reflects a progressive decline in both central and peripheral function that limits performance under sustained or high-intensity conditions [1]. As exercise continues, neurotransmitter balance in the central nervous system shifts—dopamine levels fall and serotonin activity rises—leading to reduced motor cortical drive and diminished voluntary muscle recruitment [2]. At the same time, intramuscular phosphocreatine and glycogen stores become depleted, forcing adenosine triphosphate (ATP) production toward anaerobic glycolysis [3]. The resulting accumulation of lactate, inorganic phosphate, and ammonia lowers myoplasmic pH, inhibits key glycolytic enzymes, and disrupts calcium release and uptake by the sarcoplasmic reticulum [4]. These alterations slow actin–myosin cross-bridge cycling and reduce contractile force [5]. Concurrently, elevated reactive oxygen species generated during intense contraction oxidize proteins and lipids, further impairing muscle function [6]. Recovery therefore depends on the efficient clearance of these metabolites—via enhanced blood flow and oxidative pathways—and on the activation of cellular repair and antioxidant mechanisms to restore ionic balance and replenish energy reserves.

In recent years, more and more evidence has shown that the gut microbiota plays a key role in regulating host energy metabolism and adapting to exercise [7,8]. Through fermentation of non-digestible carbohydrates, intestinal bacteria generate short-chain fatty acids (SCFAs)—principally acetate, propionate, and butyrate—which the liver can convert into glucose and which muscle tissue can oxidize for ATP production [9,10]. In addition to serving as metabolic substrates, SCFAs activate AMP-activated protein kinase (AMPK) and upregulate the expression of peroxisome proliferator-activated receptor γ coactivator-1α (PGC-1α), as demonstrated in previous animal studies [11], subsequently promoting mitochondrial biogenesis, enhancing lipid oxidation, and reducing oxidative damage, as reported by experimental studies [12]. Together, these mechanisms contribute to greater endurance capacity and more rapid recovery following exercise.

Among the factors that shape gut microbial communities, probiotic supplementation has emerged as a targeted approach to harness microbiota-mediated benefits [13]. Probiotics—defined by the World Health Organization and the Food and Agriculture Organization as live microorganisms which, when administered in adequate amounts, confer health benefits on the host—are well established for promoting intestinal barrier integrity, supporting digestive function, and modulating immune responses [14]. In addition, both animal models and human trials have demonstrated that selected probiotic strains accelerate the clearance of exercise-induced metabolites, enhancing blood lactate removal and reducing post-exercise ammonia accumulation, which together maintain acid/base balance and delay the onset of peripheral fatigue [15,16]. Probiotics significantly enhance endurance and recovery by strengthening mucosal defenses [17], producing bioactive compounds such as short-chain fatty acids, and improving nutrient absorption and mitochondrial efficiency [8]. However, the functional outcomes of probiotic administration vary substantially with strain identity, dosage, and duration of supplementation [18]. Therefore, identifying strain-specific effects and optimizing supplementation regimens are essential for effectively integrating probiotics into strategies aimed at enhancing exercise performance and recovery.

Live probiotic formulations are constrained by production and storage requirements and may be inactivated by host-specific factors within the gastrointestinal tract [19]. In contrast, inactivated or heat-treated probiotic preparations offer improved stability and safety [20]. Heat inactivation preserves key cell-surface components, such as lipoteichoic acids and peptidoglycans, which continue to engage host pattern-recognition receptors [21]. This interaction enhances mucosal barrier integrity and modulates immune responses without reliance on microbial viability. The International Scientific Association for Probiotics and Prebiotics has introduced the term “postbiotics” to encompass these inactivated microorganisms and their components, defined as non-viable microbial cells or cell fractions that, when administered in adequate amounts, confer health benefits [22]. Recent studies indicate that both heat-killed probiotics and postbiotic preparations can match live probiotics in improving exercise performance and attenuating exercise-induced inflammatory responses [23].

*Lacticaseibacillus paracasei* is a Gram-positive, facultatively anaerobic, non-spore-forming lactic acid bacterium within the family Lactobacillaceae. Strains of this species exhibit strong tolerance to gastric acid and bile salts, enabling their survival in the upper gastrointestinal tract and adhesion to the intestinal epithelium [24]. They have been shown to modulate both innate and adaptive immune responses, competitively inhibit enteric pathogens, and produce bioactive metabolites—such as exopolysaccharides and bacteriocins [25]. Moreover, *L. paracasei* strains can influence host lipid metabolism and enhance intestinal barrier integrity [26]. Previous studies have shown that supplementation with equal doses of live or heat-treated *L. paracasei* PS23 for six weeks significantly attenuated muscle strength loss after injury and reduced circulating markers of muscle damage and inflammation [27]. However, these benefits have not been evaluated for the NB23 strain. Therefore, the present study employed swimming models to assess the effects of live versus heat-treated *L. paracasei* NB23 on endurance performance, blood lactate clearance, and serum creatine kinase activity, with the aim of validating its anti-fatigue and exercise-performance-enhancing potential.

## 2. Materials and Methods

### 2.1. Sample Preparation

*Lacticaseibacillus paracasei* NB23 was isolated from a plant-based fermented product and identified as a Gram-positive lactic acid bacterium. The strain used in this study was deposited at the Bioresource Collection and Research Center (BCRC), Food Industry Research and Development Institute, Hsinchu, Taiwan, under the accession number BCRC 911201 on 4 October 2023.

For cultivation, NB23 was grown anaerobically in Lactobacilli MRS broth (Difco Laboratories, Detroit, MI, USA) at 37 °C for 24 h to reach the stationary phase. The cultured cells were then centrifuged at 3300× *g* for 10 min at 4 °C, washed twice with sterile saline, and resuspended in saline. The resulting cell suspensions were adjusted to designated concentrations and stored at −80 °C for subsequent use.

### 2.2. Experimental Design

**Figure 1:**
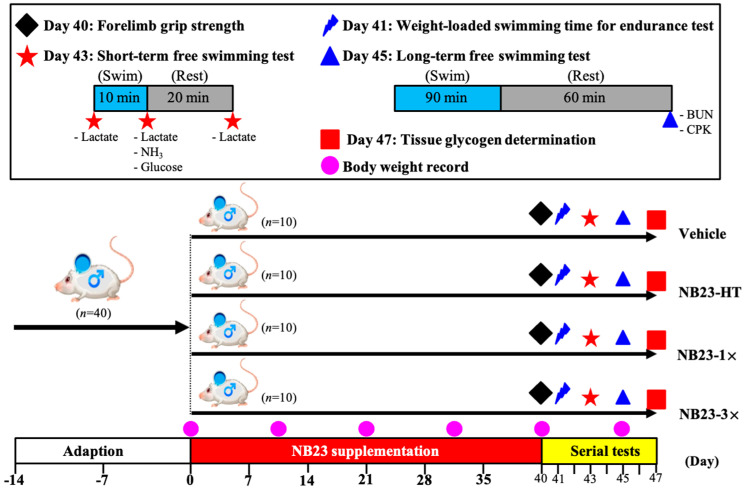
Experimental design.

Forty six-week-old male ICR mice were obtained from BioLASCO Taiwan (Yi-Lan Breeding Center, Yilan County, Taiwan). All procedures were conducted in accordance with a protocol approved by the Institutional Animal Care and Use Committee of National Taiwan Sport University (IACUC-11316). Animals were housed in a temperature-controlled room (22 ± 2 °C) with 60–70% humidity under a 12 h light/12 h dark cycle, and provided with a standard laboratory chow (No. 5001; PMI Nutrition International, Brentwood, MO, USA. The dietary composition information can be referred to at https://www.labdiet.com/product/detail/5001-laboratory-rodent-diet (accessed on 9 March 2022)) and distilled water ad libitum. The dosage of NB23 was modified from previous studies [15,28,29]. After a two-week acclimation period with unrestricted access to food and water, the mice were randomly assigned to different treatment groups using a computer-generated random number table following body weight stratification (*n* = 10 per group) to receive daily oral gavage for four weeks as follows: (1) vehicle (0 CFUs/human/day), (2) NB23–HT (3 × 10^10^ cells/human/day), (3) NB23–1× (1 × 10^10^ CFUs/human/day), (4) NB23–3× (3 × 10^10^ CFUs/human/day). We recorded the body weight, water consumption, and food intake each week. To calculate food and water intake, we administered a fixed amount of food and water, then weighed the remaining feed and water and divided it by the number of mice and the number of days to obtain the average food and water intake per mouse per day. The study design is summarized in Figure 1. To minimize potential bias during the experimental procedures, a single-blind approach was employed: the investigators responsible for administering treatments were aware of group allocation, but all outcome assessments, including sample collection, biochemical assays, and data analysis, were performed by researchers blinded to the treatment conditions.

### 2.3. Forelimb Grip Strength

Forelimb grip strength was assessed using a low-force testing apparatus (Model RX-5; Aikoh Engineering, Nagoya, Japan). Following vehicle or NB23 supplementation, each mouse was held by the tail to allow its body to hang freely while its forepaws gripped a stainless-steel rod (2 mm diameter, 7.5 cm length). A consistent, gentle horizontal pull was then applied in the opposite direction until the animal released the rod. This procedure was performed in ten successive trials, and the highest force registered by the built-in sensor was recorded as the maximum grip strength [30].

### 2.4. Swimming Exercise Performance Test

A weight-loaded swimming exhaustion test was conducted as previously described [31]. A lead weight equal to 5% of each mouse’s body weight was secured to the base of the tail, and the animals were placed individually into a water tank maintained at 27 ± 1 °C. Mice were required to swim until they exhibited loss of coordinated movement or were unable to resurface within 7 s. The interval from immersion to exhaustion was recorded as the swimming endurance time.

### 2.5. Determination of Fatigue-Associated Serum Biomarkers

We evaluated the effect of NB23 supplementation on biochemical markers and physiological states associated with post-exercise fatigue by swimming without weight loading; all mice were fasted for 12 h before each blood draw to reflect their true physiological adaptation to the exercise intervention. On day 43 after NB23 intervention, serum lactate, ammonia (NH_3_), and glucose levels were ascertained before swimming and after 10 min of swimming and 20 min of rest by submandibular blood collection. On day 45 after NB23 intervention, we also collected blood for analysis of blood urinary nitrogen (BUN) and creatine kinase (CK) after 90 min of swimming and 60 min of rest. Serum was collected from all blood samples by centrifugation at 1500× *g* for 15 min at 4 °C and was measured using an automatic analyzer (Hitachi, Tokyo, Japan, Hitachi 7060) [32].

### 2.6. Clinical Biochemical Profiles

On day 47, after 4 weeks of NB23 intervention, thirty min after the last supplementation, all mice were euthanized by 95% CO_2_, and we collected blood samples via cardiac puncture immediately. After centrifugation to collect serum, the clinical biochemical variables, including aspartate aminotransferase (AST), alanine transaminase (ALT), albumin, triglycerides (TGs), blood urea nitrogen (BUN), creatinine, uric acid (UA), total protein (TP), CK, and glucose were measured using an autoanalyzer (model 717, Hitachi, Tokyo, Japan).

### 2.7. Visceral Tissue Weight, Histology Staining, and Glycogen Determination

Following euthanasia, the mice were dissected to excise and weigh the liver, kidneys, heart, lungs, skeletal muscles, epididymal fat pads (EFPs), and brown adipose tissue (BAT). Portions of muscle and liver were then snap-frozen in liquid nitrogen and stored at −80 °C for subsequent determination of glycogen content, performed as previously described [33].

### 2.8. Statistical Analysis

All data are expressed as mean ± SD for *n* = 10 mice per group, and the statistical analysis software used was SAS 9.0 (SAS Inst., Cary, NC, USA); we used one-way analysis of variance (ANOVA) to measure statistical differences among groups. Differences between groups were analyzed by one-way ANOVA using Duncan’s post hoc test, and *p* values < 0.05 were considered significant.

## 3. Results

### 3.1. Effects of Lacticaseibacillus paracasei Supplementation on Body Weight and Food Intake

Body weight, food intake, and water consumption were measured weekly throughout the 42-day intervention (Table 1) to evaluate the effects of NB23 supplementation on growth and general health. Baseline values were comparable across all groups. During the study, all cohorts exhibited a progressive increase in body weight, and mean daily food and water intakes did not differ significantly between treatments, indicating that neither live nor heat-inactivated NB23 affected feeding or hydration. Beginning in week 5, however, mice receiving NB23–HT, NB23–1×, or NB23–3× displayed significantly lower body weight compared with controls (*p* < 0.05), suggesting that prolonged NB23 administration may modulate weight gain independently of alterations in intake.

### 3.2. Effect of NB23 Supplementation on Grip Strength and Endurance Exercise Performance

**Figure 2:**
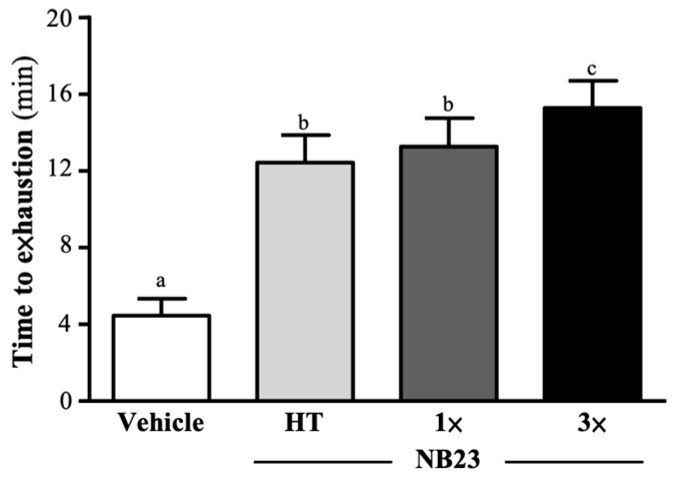
Effect of 6 weeks of NB23 supplementation on exhaustive swim time. Data expressed as mean  ±  SD for *n*  =  10 mice per group. Different superscript letters (a, b, c) indicate significant difference at *p* < 0.05.

After six weeks of NB23 supplementation, the exhaustive swim times for the vehicle, NB23–HT, NB–1× and NB23–3× groups were 3.86 ± 2.13, 5.58 ± 1.17, 9.63 ± 2.07, and 10.38 ± 1.71 min, respectively. Compared to the vehicle group, the NB23–HT, NB–1× and NB23–3× groups’ average exhaustive swim times were significantly increased 2.79-fold (*p* = 0.0404), 2.98-fold (*p* < 0.0001), and 3.43-fold (*p* < 0.0001), respectively (Figure 2).

**Figure 3:**
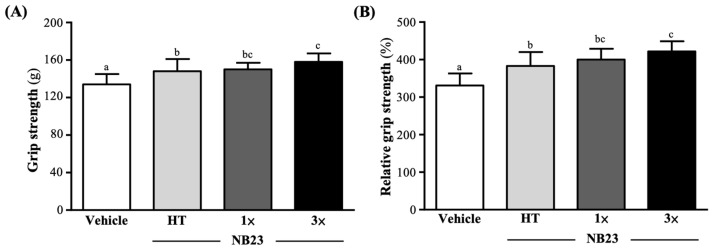
Effect of 6 weeks of NB23 supplementation on (**A**) absolute forelimb grip strength and (**B**) relative forelimb grip strength. Data expressed as mean  ±  SD for *n*  =  10 mice per group. Different superscript letters (a, b, c) indicate significant difference at *p* < 0.05.

As shown in Figure 3A, after six weeks of NB23 supplementation, the absolute forelimb grip strengths in the vehicle, NB23–HT, NB–1×, and NB23–3× groups were 134 ± 11, 148 ± 13, 150 ± 7, and 158 ± 9 (g), respectively. Compared with the vehicle group, the absolute grip strengths of the NB23–HT, NB–1×, and NB23–3× groups increased significantly: 1.11-fold (*p* = 0.0031), 1.13-fold (*p* = 0.0008), and 1.18-fold (*p* < 0.0001), respectively. We normalized by mouse body weight to calculate relative grip strength (%) and found that the NB23–HT, NB–1×, and NB23–3× supplementation groups’ relative grip strengths were also significantly greater than the vehicle group by 1.16-fold (*p* = 0.0031), 1.21-fold (*p* = 0.0008), and 1.27-fold (*p* < 0.0001) (Figure 3B).

### 3.3. Effect of NB23 Supplementation on Serum Lactate Levels After 10 Min Swim Test

After six weeks of supplementation, the mice underwent a 10 min forced swim followed by a 20 min recovery to evaluate serum lactate dynamics (Table 2). There was no significant difference in lactate levels between groups before swimming. After 10 min of swimming, the serum lactate levels of mice in the vehicle, NB23–HT, NB–1×, and NB23–3× groups were 7.37 ± 0.58, 5.27 ± 0.39, 5.03 ± 0.56, and 4.77 ± 0.46 (mmol/L), respectively. The NB23–HT, NB–1×, and NB23–3× groups’ lactate levels were significantly decreased by 28.53% (*p* < 0.0001), 31.84% (*p* < 0.0001), and 35.31% (*p* < 0.0001), respectively. Before and after 10 min of swimming, the lactate production rates were determined based on the serum lactate concentration in the vehicle, NB23–HT, NB–1×, and NB23–3× groups were 2.31 ± 0.34, 1.64 ± 0.17, 1.56 ± 0.24, and 1.47 ± 0.13, respectively. Compared with vehicle group, the NB23–HT, NB–1×, and NB23–3× groups’ values were significantly lower by 9.22% (*p* < 0.0001), 32.80% (*p* < 0.0001), and 36.54% (*p* < 0.0001), respectively.

The vehicle, NB23–HT, NB–1×, and NB23–3× groups’ blood lactate levels after 20 min rest following the swimming were 6.46 ± 0.57, 4.24 ± 0.47, 4.00 ± 0.59, and 3.73 ± 0.34 mmol/L, respectively. Compared with the vehicle group, the NB23–HT, NB–1× and NB23–3× groups’ levels were significantly lower than those of the vehicle group by 34.30% (*p* < 0.0001), 38.14% (*p* < 0.0001), and 42.27% (*p* < 0.0001), respectively. The clearance rates in the vehicle, NB23–HT, NB–1×, and NB23–3× groups were 0.12 ± 0.06, 0.19 ± 0.06, 0.21 ± 0.06, and 0.22 ± 0.06, respectively. Compared with vehicle group, the NB23–HT, NB–1×, and NB23–3× groups’ values were significantly increased 1.59-fold (*p* = 0.0150), 1.68-fold (*p* = 0.0046), and 1.76-fold (*p* = 0.0021) (*p* < 0.05).

### 3.4. Effect of NB23 Supplementation on Fatigue-Related Indexes After the 10 Min Swim Test or a 90 Min Swim Test and 60 Min Rest

**Figure 4:**
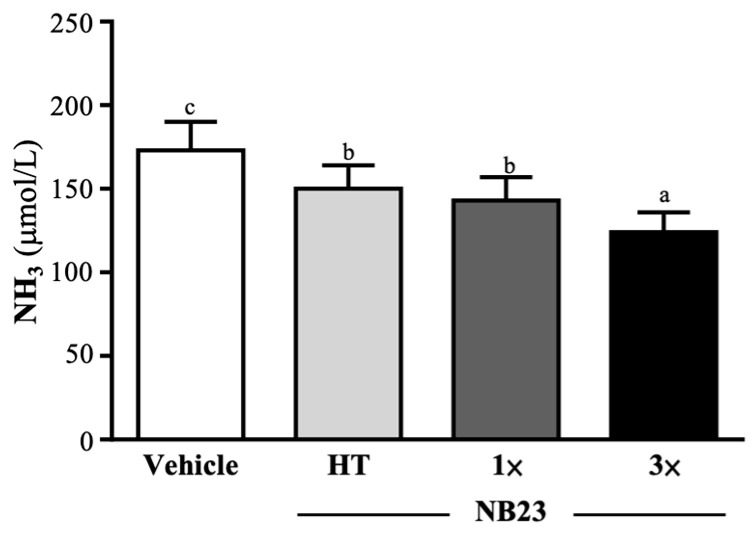
Effect of 6 weeks of NB23 supplementation on serum NH_3_ levels. Data expressed as mean  ±  SD for *n*  =  10 mice per group. Different superscript letters (a, b, c) indicate significant difference at *p* < 0.05.

Before the 10 min swim test, the vehicle, NB23–HT, NB–1×, and NB23–3× groups’ serum ammonia levels were 73 ± 3, 75 ± 4, 74 ± 5, and 73 ± 4 µmol/L, respectively; there was no significant difference between the groups. The vehicle, NB23–HT, NB–1×, and NB23–3× groups’ serum ammonia levels were 173 ± 17, 150 ± 14, 143 ± 14, and 124 ± 12 µmol/L, respectively, after the 10 min swim test. The NB23–HT, NB–1×, and NB23–3× groups’ levels were significantly decreased by 13.39% (*p* = 0.0011), 17.43% (*p* < 0.0001), and 28.03% (*p* < 0.0001), respectively, compared with vehicle group (Figure 4).

**Figure 5:**
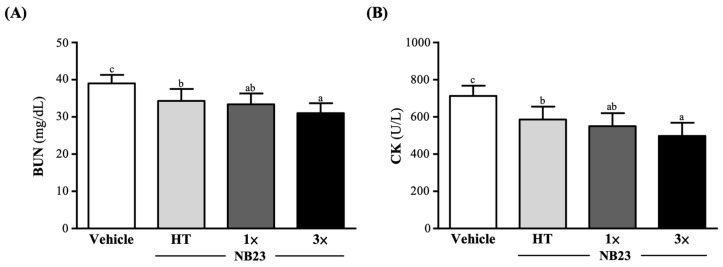
Effect of 6 weeks of NB23 supplementation on (**A**) BUN and (**B**) CK. Data expressed as mean  ±  SD for *n*  =  10 mice per group. Different superscript letters (a, b, c) indicate significant difference at *p* < 0.05. BUN, blood urea nitrogen; CK, creatine kinase.

To assess fatigue-related protein catabolism and muscle damage, serum BUN and CK levels were assessed 60 min after 90 min of non-weight-bearing swimming. Before the swim test, the vehicle, NB23–HT, NB–1×, and NB23–3× groups’ serum blood urea nitrogen (BUN) concentrations were 23.1 ± 3.4 23.1 ± 3.8, 23.2 ± 3.6, and 23.0 ± 3.4 (mg/dL), respectively; there was no significant difference between the groups. After 90 min of non-weight-bearing swimming then a 60 min rest, the BUN concentrations in the vehicle, NB23–HT, NB–1×, and NB23–3× supplementation groups were 39.0 ± 2.3, 34.3 ± 3.2, 33.4 ± 2.9, and 31.0 ± 2.7 (mg/dL), respectively. Compared to the vehicle group, BUN levels were significantly reduced by 11.89% (*p* = 0.0007), 14.20% (*p* < 0.0001), and 20.41% (*p* < 0.0001) in the NB23–HT, NB–1×, and NB23–3× groups, respectively (Figure 5A).

The CK activity indexes showed no significant difference between the vehicle, NB23–HT, NB–1×, and NB23–3× supplementation groups, at 213 ± 22, 209 ± 28, 215 ± 19, and 220 ± 31 µmol/L, respectively. The CK activity indexes in the vehicle, NB23–HT, NB–1×, and NB23–3× groups were 713 ± 55, 586 ± 69, 550 ± 70, and 498 ± 71 (U/L), respectively, after 90 min of non-weight-bearing swimming then a 60 min rest. Compared with the vehicle group, the NB23–HT, NB–1×, and NB23–3× group indexes significantly decreased by 17.78% (*p* = 0.0001), 22.82% (*p* < 0.0001), and 30.12% (*p* < 0.0001), respectively (Figure 5B).

### 3.5. Effect of NB23 Supplementation on Biochemical Profiles at End of Study

To evaluate the safety of six weeks of NB23 supplementation, we measured a panel of serum biochemical markers (Table 3). All parameters remained within established normal ranges, with no statistically significant differences observed among the treatment groups.

### 3.6. Effect of NB23 Supplementation on Liver and Muscle Glycogen

**Figure 6:**
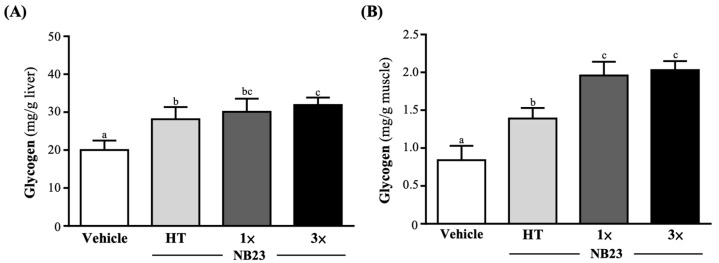
Effect of 6 weeks of NB23 supplementation on (**A**) liver and (**B**) muscle. Data expressed as mean  ±  SD for *n*  =  10 mice per group. Different superscript letters (a, b, c) indicate significant difference at *p* < 0.05.

As shown on Figure 6A, the vehicle, NB23–HT, NB–1×, and NB23–3× groups’ liver glycogen levels were 20.03 ± 2.48, 28.15 ± 3.22, 30.10 ± 3.47, and 31.88 ± 1.97 mg/g liver, respectively. The NB23–HT, NB–1×, and NB23–3× groups’ levels were significantly improved compared to the vehicle group by 1.41-fold (*p* = 0.0051), 1.50-fold (*p* < 0.0001), and 1.59-fold (*p* < 0.0001), respectively. The muscle glycogen levels in the vehicle, NB23–HT, NB–1×, and NB23–3× groups were 0.84 ± 0.19, 1.39 ± 0.14, 1.96 ± 0.18, and 2.03 ± 0.15 mg/g muscle, respectively. Compared with vehicle group, the NB23–HT, NB–1×, and NB23–3× group levels were significantly increased 1.65-fold (*p* < 0.0001), 2.34-fold (*p* < 0.0001), and 2.42-fold (*p* < 0.0001), respectively (Figure 6B).

### 3.7. General Characteristics of Mice and Histopathology of Tissues with NB23 Supplementation for Six Weeks

After six consecutive weeks of supplementation with NB23, there was no significant difference in absolute and relative liver, muscle, kidney, heart, lung, BAT, and cecum weight, respectively. However, after six weeks of supplementation, the NB23–HT, NB–1×, and NB23–3× groups showed significantly decreased absolute EFP weights compared to the vehicle group, lower by 24.18% (*p* = 0.0107), 23.58% (*p* = 0.0126), and 31.04% (*p* = 0.0014), respectively. In terms of relative EFP weight, the NB23–HT, NB–1×, and NB23–3× groups’ values were significantly lower than the vehicle group by 22.70% (*p* = 0.0197) and 27.70% (*p* = 0.0055), respectively (Table 4).

**Figure 7:**
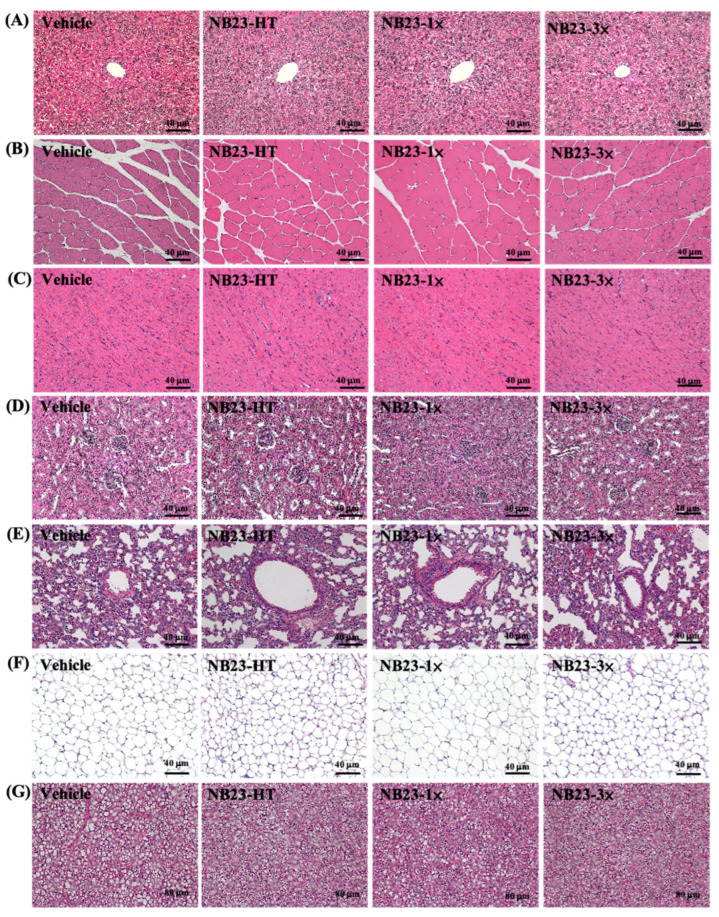
Effect of NB23 supplementation on (**A**) liver, (**B**) muscle, (**C**) kidney, (**D**) heart, (**E**) lung, (**F**) adipocyte tissue, (**G**) BAT in mice. H&E stain, magnification: 200×; bar, 40 μm; BAT magnification: 100×; bar, 80 μm. EFP, epididymal fat pad; BAT, brown adipose tissue.

In addition, we performed histological evaluations of the liver, muscle, heart, kidney, lung, EFP, and BAT of mice, and no abnormalities or pathological changes were found in any group (Figure 7). Therefore, we believe that supplementation with live or heat-treated of NB23 does not cause any harm.

## 4. Discussion

The present study demonstrates that six weeks of supplementation with *Lacticaseibacillus paracasei* NB23, whether administered as live bacteria (1 × 10^10^ or 3 × 10^10^ CFUs per human per day) or as a heat-inactivated postbiotic preparation, produces a series of coordinated physiological adaptations. These physiological adaptations may contribute to improved endurance capacity, muscle strength, and energy substrate utilization, as well as attenuation of biochemical and histopathological markers associated with exercise-induced fatigue. Importantly, no significant alterations in voluntary food or water intake nor indications of organ toxicity were observed. Future studies are warranted to confirm and further explore these findings.

Probiotic and postbiotic interventions attenuate diet-induced adiposity by reprogramming host energy metabolism and reinforcing gut barrier function [34]. In high-fat-diet models, supplementation enriches SCFA-producing taxa (e.g., Akkermansia, Bacteroides), restoring the Firmicutes/Bacteroidetes balance and elevating acetate, propionate, and butyrate levels, which activate GPR41/GPR43 and AMP-activated protein kinase (AMPK) to suppress SREBP-1c-mediated lipogenesis and upregulate CPT1-driven fatty-acid oxidation [35,36,37]. Concurrently, butyrate acts as an HDAC inhibitor, epigenetically promoting PGC-1α expression and mitochondrial biogenesis in muscle and adipose tissue [38]. Microbial bile salt hydrolase activity further remodels the bile acid pool to attenuate FXR signaling, thereby enhancing thermogenesis and lipid catabolism [39]. At the same time, cell-surface ligands retained in heat-killed preparations (lipoteichoic acids, peptidoglycan) engage TLR2/6 and NOD2 to strengthen tight junctions (ZO-1, occludin), reduce lipopolysaccharide translocation, and dampen chronic inflammation (lower TNF-α, IL-6), which restores insulin sensitivity and limits adipocyte hypertrophy [40]. In a previous study, mice were fed a high-fat diet for eight weeks and received a daily oral dose of *L. paracasei* AO356 at either 1 × 10^7^ CFU per animal or 1 × 10^8^ CFU per dose. This treatment significantly downregulated key lipogenic genes—Srebp1c, Pparγ, Fas, C/ebpα, and Fabp4—while upregulating thermogenic and mitochondrial-biogenesis genes such as Ucp1, Cpt1, Pgc1α, Cidea, and Prdm16. As a result, the treated mice exhibited reduced body weight and fat mass, smaller adipocyte size, and increased skeletal muscle fiber diameter [41]. In NB23-supplemented mice, we observed comparable chow and water intake alongside a modest reduction in epididymal fat pad mass and moderated body weight gain from week 5 onwards, suggesting a potential shift in energy partitioning toward oxidation rather than storage. Concurrent increases in hepatic and muscle glycogen, faster clearance of lactate and ammonia, and enhanced endurance performance were also observed, collectively indicating that NB23 supplementation may support beneficial metabolic adaptations and improved exercise capacity. However, further direct investigations into underlying mechanisms are necessary to substantiate these preliminary observations.

By alleviating lipid-induced metabolic stress and dampening chronic inflammation, NB23 creates a physiological milieu that favors enhanced glucose clearance and storage. Although we did not directly assess insulin-receptor signaling, the pronounced increases in hepatic (41–59%) and muscle (65–142%) glycogen (Figure 6A,B) closely mirror the effects reported after four weeks of *Lactiplantibacillus plantarum* PL-02 or *Bifidobacterium longum* OLP-01 supplementation, which activate AMPK–PGC-1α signaling to promote mitochondrial biogenesis and GLUT4-mediated glucose uptake [31,42]. In those models, AMPK phosphorylation upregulates both the expression and translocation of GLUT4 to the sarcolemma, while PGC-1α drives mitochondrial proliferation and induces glycogen synthase and other glycogenic enzymes [43]. These coordinated responses enhance glucose uptake from the circulation and its incorporation into glycogen. Butyrate produced by NB23 may inhibit histone deacetylases, leading to epigenetic modifications that enhance transcription of genes encoding oxidative myofibrillar proteins and key glycolytic enzymes such as hexokinase II. This mechanism could underlie a shift in muscle fiber composition toward a greater proportion of mitochondria-rich, fatigue-resistant fibers, although we did not directly measure HDAC activity in this study [44]. The resulting structural remodeling, together with expanded intracellular glycogen reserves, enlarges the ATP buffer available to working muscle and sustains high rates of oxidative phosphorylation during prolonged exercise bouts [45]. Furthermore, these augmented glycogen stores likely accelerate post-exercise recovery by facilitating rapid ATP resynthesis [46]. Collectively, these metabolic and structural adaptations provide a robust mechanistic rationale for the delayed onset of fatigue observed in NB23-supplemented animals. These metabolic and structural adaptations establish the bioenergetic and contractile foundation necessary for enhanced performance. By expanding intracellular glycogen stores and promoting a shift toward mitochondria-rich, fatigue-resistant fibers, NB23-supplemented muscle is better equipped to sustain ATP turnover and maintain force generation under prolonged load [47]. Consequently, these preconditioning effects translate directly into functional gains: NB23 supplementation increases exhaustive-swim time and absolute forelimb grip strength, with relative (body-weight-normalized) strength improvements (Figure 2 and Figure 3A,B). Such ergogenic outcomes closely mirror those reported for supplementation with probiotics for multi-week probiotic regimens, with similarly extended time to exhaustion, increased muscle, and augmented force output [48].

Existing research into the anti-fatigue properties of *L. paracasei* has primarily focused on its modulation of the hypothalamic–pituitary–adrenal (HPA) axis, demonstrating reductions in circulating cortisol and improvements in neurocognitive function and overall well-being in patients with chronic fatigue syndrome [49,50]. In the context of exercise-induced fatigue, both viable and heat-inactivated preparations of *L. paracasei* have been shown to accelerate post-exercise recovery of muscle strength and to attenuate rises in creatine kinase, myoglobin, and malondialdehyde, thereby delaying fatigue onset [27]. Nevertheless, the effects of *L. paracasei* on endurance performance and its influence on key biochemical markers of exercise fatigue have not been examined. In the present study, we report that six weeks of supplementation with *L. paracasei* NB23 significantly attenuates post-exercise elevations in blood lactate, ammonia, creatine kinase, and blood urea nitrogen (Table 2, Figure 4 and Figure 5A,B). These biochemical parameters are likewise recognized to increase with greater exercise duration or intensity, an effect that correlates with diminished exercise capacity and the accumulation of fatigue-related by-products [51]. During intense exercise, anaerobic glycolysis converts glucose to pyruvate and then to lactate, with concomitant proton release lowering muscle and blood pH and inhibiting glycolytic enzymes and calcium handling, thereby impairing contractile function and promoting fiber damage [52]. Simultaneously, ATP resynthesis via AMP deaminase generates ammonia, which accumulates in skeletal muscle and, if not efficiently removed by hepatic urea cycle activity (BUN), can exacerbate both peripheral and central fatigue [53]. Probiotic supplementation offers a multifaceted mitigation strategy: specific strains accelerate the microbial conversion of exercise-derived lactate into SCFAs such as propionate and butyrate, which are subsequently transformed into acetyl-CoA to fuel the tricarboxylic acid cycle and sustain ATP production [54], thus lowering circulating lactate levels and providing an additional energy source. Concurrently, probiotics enhance barrier integrity by upregulating tight-junction proteins and inhibiting bacterial urease activity, reducing luminal ammonia generation and systemic translocation, which leads to lower post-exercise blood ammonia concentrations [55,56]. Animal studies with *Ligilactobacillus salivarius* SA-03 have substantiated these mechanisms, demonstrating significant reductions in exercise-induced lactate, ammonia, BUN, and creatine kinase, alongside improved blood glucose availability [57]. Our findings with NB23 parallel these effects, confirming that targeted probiotic intervention can both expedite metabolite clearance and reinforce energy homeostasis during and after prolonged exertion.

Importantly, NB23’s ergogenic and anti-fatigue effects were equally evident in its heat-inactivated form, supporting the feasibility of postbiotic applications. This suggests that bacterial viability is not essential for functional benefits, and that key bioactive components may be heat-stable. Previous research has identified several such components, including lipoteichoic acids (LTAs), peptidoglycans, surface-layer proteins, and exopolysaccharides (EPSs), which are known to retain immunomodulatory or metabolic signaling properties after heat treatment. These molecules can interact with pattern-recognition receptors (e.g., TLR2) in the host, triggering downstream effects that influence energy metabolism, inflammation, and fatigue recovery. In particular, heat-treated Lactobacillus strains have been shown to enhance exercise performance and modulate fatigue-related pathways in studies [27,29]. Moreover, the use of heat-inactivated probiotics may offer advantages in terms of formulation stability, safety for immunocompromised individuals, and regulatory compliance [58]. These findings support the translational potential of *L. paracasei* NB23 not only as a live probiotic, but also as a functional postbiotic agent. Both live and postbiotic NB23 preparations preserve normal clinical-chemistry parameters (Table 3) and cause no histopathological alterations in the liver, skeletal muscle, heart, kidney, lung, white adipose, or brown adipose tissues (Figure 7), confirming broad biocompatibility. These findings are consistent with a mechanistic framework, supported by prior studies, in which *L. paracasei* strains remodel the gut microbiota to increase SCFA production, thereby activating AMPK–PGC-1α signaling and associated epigenetic pathways that promote mitochondrial biogenesis and glycogen storage [59]. Such strains have also been shown to upregulate monocarboxylate transporters and urea-cycle enzymes, accelerating lactate and ammonia clearance, as well as to enhance barrier integrity and reduce inflammation, which preserves hepatic detoxification capacity. Moreover, probiotic interventions can influence adipocyte metabolism suppressing lipogenesis and stimulating fatty-acid oxidation to improve body composition. While our study did not directly measure these pathways, the improvements in endurance, strength, and metabolic markers we observed align with these documented mechanisms and provide a rationale for further targeted investigations. This integrated network of metabolic, structural, and immunological adaptations underpins the marked improvements in endurance and strength observed without adverse health effects.

To translate these findings, future studies should employ comprehensive multi-omics approaches—combining metagenomics, metabolomics and host transcriptomics—to pinpoint the NB23-derived metabolites and host pathways (e.g., mTOR/IGF-1, HDAC targets) driving the ergogenic phenotype; conduct dose-ranging and formulation trials in humans to establish optimal live versus postbiotic regimens and ensure tolerability; and perform randomized, placebo-controlled studies measuring VO_2_max, time to exhaustion, strength gains, recovery kinetics, body-composition changes, and safety endpoints in athletes under structured training. Ultimately, personalized probiotic strategies informed by baseline microbiome profiles and genetic variants in SCFA receptors (GPR41/43) and metabolic sensors may maximize responsiveness and support evidence-based integration of *L. paracasei* NB23 into sports-nutrition protocols for performance enhancement, accelerated recovery, and metabolic health.

This study has several limitations. First, although significant physiological changes were observed, the findings are based on an animal model, and caution should be exercised when extrapolating to human applications. Second, while multiple fatigue-related biomarkers were analyzed, comprehensive metabolic profiling and molecular pathway validation (e.g., gene or protein expression analysis of AMPK/PGC-1α signaling) were not performed. Third, spontaneous physical activity and individual lactate thresholds were not assessed, which could have provided further insight into exercise adaptation mechanisms. Fourth, serum rather than plasma was used for biochemical analyses due to sample volume constraints, which may introduce variability in certain measurements. Fifth, although data distributions were visually examined and group sizes were balanced, formal statistical tests for normality and homogeneity of variances (e.g., Shapiro–Wilk and Levene’s test) were not performed, which may influence the interpretability of parametric results. Future studies incorporating broader mechanistic analyses, diverse exercise models, and translational clinical trials are warranted to strengthen and extend these findings.

## 5. Conclusions

Six weeks of *L. paracasei* NB23 supplementation—administered live (1 × 10^10^ or 3 × 10^10^ CFUs/human/day) or heat-treated—significantly enhanced murine exercise endurance and forelimb grip strength, expanded hepatic and muscle glycogen stores, and attenuated post-exercise elevations in lactate, ammonia, BUN, and CK, all without altering food intake or inducing organ toxicity. The equivalence of live and postbiotic NB23 in both efficacy and safety underscores its translational promise as a stable, well-tolerated intervention to mitigate exercise-induced fatigue and support performance.

## Figures and Tables

**Table 1:** Changes in body weight and food intake among experimental groups during the study period.

Characteristics	Vehicle	NB23–HT	NB23–1×	NB23–3×
Initial BW (g)	34.6 ± 1.5 ^a^	34.7 ± 0.5 ^a^	34.7 ± 1.3 ^a^	34.7 ± 0.7 ^a^
1st wk BW	35.5 ± 1.2 ^a^	35.3 ± 0.8 ^a^	34.6 ± 1.2 ^a^	34.8 ± 1.1 ^a^
2nd wk BW	36.5 ± 1.6 ^b^	35.9 ± 1.2 ^b^	35.4 ± 1.7 ^ab^	34.5 ± 1.0 ^a^
3rd wk BW	38.1 ± 1.4 ^c^	36.4 ± 1.2 ^b^	36.1 ± 1.8 ^ab^	34.9 ± 1.0 ^a^
4th wk BW	38.8 ± 1.2 ^b^	37.3 ± 1.1 ^a^	36.9 ± 1.9 ^a^	36.2 ± 0.8 ^a^
5th wk BW	39.4 ± 1.2 ^b^	37.8 ± 1.4 ^a^	37.1 ± 2.2 ^a^	36.7 ± 1.0 ^a^
6th wk BW	40.4 ± 1.3 ^b^	38.8 ± 1.2 ^a^	37.7 ± 2.2 ^a^	37.5 ± 1.0 ^a^
7th wk BW	39.6 ± 1.6 ^b^	38.8 ± 1.1 ^a^	37.9 ± 2.1 ^a^	37.9 ± 0.9 ^a^
Final BW (g)	40.1 ± 1.6 ^b^	39.2 ± 1.1 ^b^	37.7 ± 2.2 ^a^	38.3 ± 1.1 ^ab^
Water intake (mL/mouse/day)	7.9 ± 0.6 ^a^	7.9 ± 0.8 ^a^	7.8 ± 0.7 ^a^	7.9 ± 0.9 ^a^
Diet intake (g/mouse/day)	6.7 ± 0.8 ^a^	6.8 ± 0.8 ^a^	6.8 ± 0.4 ^a^	6.8 ± 0.3 ^a^

The experimental animals were randomly divided into four groups, with 10 mice in each group: (1) Vehicle, (2) NB23-HT, (3) NB23–1×, (4) NB23–3×. All data are presented as mean ± SD. Different superscript letters (a, b, c) indicate significant difference at *p* < 0.05. The standard chow diet used (Chow 5001) provided 3.36 kcal/g of energy.

**Table 2:** Effect of NB23 on lactate levels.

Time Point	Vehicle	NB23-HT	NB23-1×	NB23-3×
	Lactate (mmol/L)			
Before swimming (A)	3.24 ± 0.54 ^a^	3.23 ± 0.28 ^a^	3.27 ± 0.37 ^a^	3.26 ± 0.28 ^a^
After swimming (B)	7.37 ± 0.58 ^c^	5.27 ± 0.39 ^b^	5.03 ± 0.56 ^a^	4.77 ± 0.46 ^a^
After a 20 min rest (C)	6.46 ± 0.57 ^c^	4.24 ± 0.47 ^b^	4.00 ± 0.59 ^a^	3.73 ± 0.34 ^a^
Rate of lactate production and clearance				
Production rate = B/A	2.31 ± 0.34 ^c^	1.64 ± 0.17 ^b^	1.56 ± 0.24 ^a^	1.47 ± 0.13 ^a^
Clearance rate = (B − C)/B	0.12 ± 0.06 ^a^	0.19 ± 0.06 ^a^	0.21 ± 0.06 ^a^	0.22 ± 0.06 ^b^

The lactate production rate (B/A) was the value of the lactate level after exercise (B) divided by that before exercise (A). Clearance rate (B − C)/B was defined as lactate level after swimming (B) minus that after 20 min of rest (C) divided by that after swimming (B). Data are expressed as mean ±  SD (*n*  =  10 mice per group). Values in the same row with different superscript letters (a, b, c) differ significantly, *p*  <  0.05.

**Table 3:** Effect of NB23 supplementation on biochemical parameters.

Parameter	Vehicle	NB23-HT	NB23-1×	NB23-3×
AST (U/L)	93 ± 12 ^a^	95 ± 18 ^a^	95 ± 14 ^a^	94 ± 9 ^a^
ALT (U/L)	51 ± 4 ^a^	52 ± 6 ^a^	51 ± 9 ^a^	51 ± 12 ^a^
GLU (mg/dL)	204 ± 17 ^a^	205 ± 14 ^a^	204 ± 17 ^a^	205 ± 23 ^a^
CREA (mg/dL)	0.40 ± 0.02 ^a^	0.39 ± 0.02 ^a^	0.39 ± 0.02 ^a^	0.39 ± 0.03 ^a^
BUN (mg/dL)	19.2 ± 1.4 ^a^	19.3 ± 1.7 ^a^	19.2 ± 1.4 ^a^	19.2 ± 1.9 ^a^
UA (mg/dL)	1.8 ± 0.7 ^a^	1.9 ± 0.6 ^a^	1.9 ± 0.6 ^a^	1.9 ± 0.5 ^a^
TC (mg/dL)	154 ± 17 ^a^	153 ± 15 ^a^	154 ± 16 ^a^	155 ± 16 ^a^
TG (mg/dL)	157 ± 17 ^a^	158 ± 14 ^a^	158 ± 12 ^a^	157 ± 22 ^a^
ALB (g/dL)	3.29 ± 0.15 ^a^	3.28 ± 0.12 ^a^	3.27 ± 0.22 ^a^	3.29 ± 0.15 ^a^
TP (g/dL)	5.6 ± 0.2 ^a^	5.6 ± 0.1 ^a^	5.6 ± 0.3 ^a^	5.6 ± 0.1 ^a^

Data are expressed as mean ± SD (*n* = 10 mice per group). Values in the same row with the same superscript letters (a) do not differ significantly, *p*  >  0.05. AST, aspartate aminotransferase; ALT, alanine transaminase; ALB, albumin; BUN, blood urea nitrogen; CREA, creatinine; UA, uric acid; TP, total protein; TG, triacylglycerol; CK, creatine kinase.

**Table 4:** Effect of NB23 supplementation on tissue weight.

Characteristic	Vehicle	NB23-HT	NB23-1×	NB23-3×
Liver (g)	1.87 ± 0.12 ^a^	1.83 ± 0.15 ^a^	1.82 ± 0.11 ^a^	1.84 ± 0.13 ^a^
Muscle (g)	0.40 ± 0.02 ^a^	0.40 ± 0.03 ^ab^	0.40 ± 0.03 ^ab^	0.39 ± 0.02 ^b^
Kidney (g)	0.66 ± 0.08 ^a^	0.65 ± 0.06 ^a^	0.63 ± 0.05 ^a^	0.64 ± 0.04 ^a^
Heart (g)	0.19 ± 0.03 ^a^	0.19 ± 0.02 ^a^	0.19 ± 0.02 ^a^	0.19 ± 0.01 ^a^
Lung (g)	0.29 ± 0.02 ^a^	0.28 ± 0.02 ^a^	0.28 ± 0.03 ^a^	0.29 ± 0.03 ^a^
EFP (g)	0.34 ± 0.09 ^b^	0.25 ± 0.06 ^a^	0.26 ± 0.06 ^a^	0.23 ± 0.06 ^a^
BAT (g)	0.09 ± 0.02 ^a^	0.09 ± 0.02 ^a^	0.09 ± 0.02 ^a^	0.09 ± 0.02 ^a^
Cecum (g)	0.98 ± 0.09 ^a^	0.97 ± 0.14 ^a^	0.95 ± 0.13 ^a^	0.96 ± 0.07 ^a^
Relative liver weight (%)	4.68 ± 0.45 ^a^	4.68 ± 0.45 ^a^	4.83 ± 0.26 ^a^	4.82 ± 0.38 ^a^
Relative muscle weight (%)	1.00 ± 0.07 ^a^	1.01 ± 0.06 ^b^	1.06 ± 0.07 ^b^	1.02 ± 0.08 ^c^
Relative kidney weight (%)	1.64 ± 0.15 ^a^	1.65 ± 0.15 ^a^	1.67 ± 0.08 ^a^	1.67 ± 0.12 ^a^
Relative heart weight (%)	0.48 ± 0.07 ^a^	0.49 ± 0.05 ^a^	0.51 ± 0.04 ^a^	0.50 ± 0.03 ^a^
Relative lung weight (%)	0.72 ± 0.04 ^a^	0.72 ± 0.07 ^a^	0.76 ± 0.09 ^a^	0.76 ± 0.07 ^a^
Relative EFP weight (%)	0.84 ± 0.23 ^b^	0.65 ± 0.13 ^a^	0.68 ± 0.17 ^ab^	0.60 ± 0.16 ^a^
Relative BAT weight (%)	0.22 ± 0.04 ^a^	0.24 ± 0.04 ^a^	0.24 ± 0.06 ^a^	0.23 ± 0.04 ^a^
Relative cecum weight (%)	2.45 ± 0.28 ^a^	2.48 ± 0.37 ^a^	2.52 ± 0.29 ^a^	2.50 ± 0.21 ^a^

Data are expressed as mean ± SD (*n* = 10 mice per group). Values in the same row with different superscript letters (a, b, c) differ significantly, *p*  <  0.05. EFP, epididymal fat pad; BAT, brown adipose tissue.

## Data Availability

The original contributions presented in this study are included in the article. Further inquiries can be directed to the corresponding authors.
